# Optimization of nutrient management improves productivity, quality and sustainability of albino tea cultivar Baiye-1

**DOI:** 10.3389/fpls.2024.1369015

**Published:** 2024-05-02

**Authors:** Yun Zhu, Lifeng Ma, Saipan Geng, Jianyun Ruan

**Affiliations:** ^1^ Tea Research Institute, Chinese Academy of Agricultural Sciences, Hangzhou, Zhejiang, China; ^2^ Key Laboratory of Biology, Genetics and Breeding of Special Economic Animals and Plants, Ministry of Agriculture and Rural Affairs, Hangzhou, Zhejiang, China; ^3^ Xihu National Agricultural Experimental Station for Soil Quality, Ministry of Agriculture and Rural Affairs, Hangzhou, Zhejiang, China

**Keywords:** free amino acid, catechin, organic substitution, nutrient use efficiency (NUE), greenhouse gas emissions, nitrogen nutrition, albino tea cultivar

## Abstract

Proper nutrient management is crucially important to the sustainable development of tea production. Compared to normal green-leaf cultivars, albino tea cultivars produce green tea of superior quality characterized by high contents of amino acids as a result of the hydrolysis of chloroplast proteins at albinism. However, the advantage of albino tea cultivars was offset by inferior growth and yield performance because of low contents of chlorophylls and limited photosynthesis capacity. Our understanding about the nutrition characteristics of albino tea cultivars was very limited. A four-year field experiment was conducted to develop proper nutrient management for Baiye-1 to overcome its weakness of low productivity without a tradeoff in tea quality and environmental risks. The nutrient management schemes were formulated by optimizing the rate and ratio of nitrogen (N), phosphorus, potassium and magnesium together with substitution of chemical fertilizers with organic manures. The total amounts of nutrients in the optimized schemes were reduced by 25% compared to the local farmers’ practice (FP). Results showed that optimized rates and ratio of nutrients together with partial substitution of chemical fertilizers with rapeseed cake manure more considerably improved albino tea yield, the contents of free amino acids, total polyphenol and catechins relative to FP. Partial substitution of chemical fertilizers with commercial livestock manure decreased tea quality, which was likely caused by a dilution effect of increasing tea yield and decreasing N status of tea plants. Full organic substitution of chemical fertilizers by rapeseed cake manure improved tea yield and quality but had relatively low agronomic efficiency and profit. The effect of optimized nutrient management schemes was associated with the improvement of nutritional status in tea plants. The present work demonstrated that the optimization of nutrient management considerably improved albino tea yield, quality and profit while decreased the application rate of fertilizers and the intensity of greenhouse gas emissions.

## 1 Introduction

Tea (*Camellia sinensis*) is a valuable cash crop widely planted in Asia and Africa and plays important roles in increasing farmers’ income and alleviating poverty of rural areas. The tea quality is determined by internal chemical ingredients (Zhang and Ruan, 2016). In recent years, natural mutants with albino, yellow or purple young shoots have been cultivated and cherished for their high contents of components such as free amino acids, flavonoids and anthocyanins in China, Japan and Kenya (Kilel et al., 2013; Li et al., 2018; Yamashita et al., 2021). Baiye-1 (once named as Anjibaicha) is a temperature-sensitive albino tea cultivar. In spring, young shoots of Baiye-1 show periodic change from green to etiolated in the early spring and afterwards back to green colors, which is dependent upon the air temperature. At albinism the chloroplast is destructed and protease activity increases leading to the hydrolysis of proteins and high accumulation of free amino acids (Li et al., 2018). Thus, green tea processed from young shoots of albino cultivars has superior quality of strong umami taste and fresh aroma resulting from the high level of amino acids and hence higher price and economic profit. For example, it was reported that the average price of fresh young shoots of Baiye-1 was 200 − 300 Yuan kg^-1^, which was 2 − 3 times of normal varieties in Hubei province (Zeng, 2018). High benefit promoted the rapid expansion of Baiye-1, making it one of the largest clonal varieties planted in China with an area of 267 thousand hectares in 2015 and the planting area was kept increasing in the recent years (Li et al., 2020). However, the economic advantage albino tea cultivars was offset by inferior growth and yield performance due to low contents of chlorophylls and limited photosynthesis capacity (Zhang et al., 2020). According to a recent survey covering 26 counties of nine provinces, the profit per area of Baiye-1 was only 27% higher than other varieties (Professor Aiqin Jiang, personal communication). Therefore, promotion of yield while maintaining good quality in the meantime is a challenge for albino tea cultivars including Baiye-1.

Fertilization is an essential field management to ensure tea yield, quality and profit in the meantime as nutrient deficiency significantly reduces the contents of amino acids and aroma compounds in tea (Zhou et al., 2022). Proper nutrient management requires the application of right source of nutrients at the right rate (Johnston and Bruulsema, 2014). Nitrogen (N), phosphorus (P) and potassium (K) are the most popularly nutrients applied in tea plantations (Mishima et al., 2010; Ni et al., 2019). Increasing N supply stimulates the expression of genes and the activity of enzymes involved in N uptake and assimilation, and promotes the biosynthesis and accumulation of amino acids (Ruan et al., 2010; Liu et al., 2020; 2021). Application of P and K fertilizers increased the concentrations of total polyphenols, catechins, free amino acids and aroma compounds (Lin et al., 2012) (Venkatesan and Ganapathy, 2004; Ruan et al., 2013). However, recent works showed that the overuse of chemical fertilizers and improper nutrient balance in tea plantations had been a major problem (Mishima et al., 2010; Ni et al., 2019). Excessive supply of N, P and K nutrients reduces tea quality (Venkatesan and Ganapathy, 2004; Owuor et al., 2013; Ding et al., 2017; Chen et al., 2021). Furthermore, high and excessive application of synthetic N fertilizer deteriorates soil properties (Yang et al., 2018; Ma et al., 2021) and induces strong N_2_O emission (Wang et al., 2020a). Therefore, the recommendation of the right rate of fertilizers is the preconditions ensuring profitable and environmental friendly tea production with reduced greenhouse gas emissions (Liang et al., 2021; Tang et al., 2021). On the other hand, organic fertilizers such as de-oiled rapeseed cake manure and decomposed livestock and poultry excrement were important nutrient sources for tea plantations (Ni et al., 2019; Sun et al., 2021). Combining application of organic manures with chemical fertilizers is recommended as a practical solution to reduce the input of chemical fertilizers and to improve nutrient use efficiency without negative impact on tea yield and quality (Wang et al., 2020b; Huang et al., 2022; Ma et al., 2022). There were large body of information concerning nutrition characteristics and fertilization of normal green-leaf cultivars. However, to the best of our knowledge, the understanding about the nutrition characteristics of albino tea cultivars was extremely limited. Furthermore, the mechanism of high accumulation of free amino acids in Baiye-1 is different from that of normal green leaf tea cultivars of which is a result of stimulated N assimilation (Ruan et al., 2010; Liu et al., 2020; Zhang et al., 2020). There is a need to develop proper nutrient management for Baiye-1 to overcome its weakness of low productivity without a tradeoff in tea quality and environmental risks.

The present field experiment was conducted to test the effect of optimized nutrient management on yield, quality and nutrient use efficiency of Baiye-1 tea plantation. The nutrient management practices were optimized by integrating two approaches. One approach was to optimize the rates of nutrients N, P, K, Mg and their ratio by replacing the common compound NPK fertilizer of equal nutrient formular (N-P_2_O_5_-K_2_O = 15-15-15) with a specially formulated compound fertilizer (N-P_2_O_5_-K_2_O-MgO = 18-8-12-2). Another approach was to substitute chemical fertilizers fully or partly with organic manures, i.e. rapeseed cake manure or commercial livestock manure. These schemes were compared to farmer’s practices. The objective was to develop efficient nutrient management schemes for albino tea plantations towards better yield and quality, higher profit with less environmental risks.

## 2 Materials and methods

### 2.1 Field experiment

A field experiment was set up in 2016 in Boming tea plantation, Anji County, Zhejiang Province, China. The soil pH was 4.48 and the contents of organic matter, total N, available P, available K and available Mg were 14.36 mg g^-1^, 0.86 mg g^-1^, 52 mg kg^-1^, 131 mg kg^-1^ and 30 mg kg^-1^, respectively. The tea cultivar was Baiye-1 and the plants were 20 years old. Before the start of field experiment, tea plants received common compound fertilizer (N-P_2_O_5_-K_2_O = 15-15-15) together with rapeseed cake manure (N-P_2_O_5_-K_2_O = 5.8-2.7-1.5), a practice widespread in tea plantations of China according to a previous survey (Ni et al., 2019). This practice is hereafter referred to as farmers’ practice (FP) and was compared to three optimized schemes (treatments) (**Table 1**). In the first scheme referred to as RSM, the chemical compound fertilizers were fully substituted by rapeseed cake manure (RSM). In the second scheme referred to as SCF+RSM, the common compound fertilizer of equal nutrient formular was replaced by a specially formulated compound fertilizer (N-P_2_O_5_-K_2_O-MgO = 18-8-12-2) together with organic manure rapeseed cake and urea. In the third scheme referred to as SCF+LSM, the specially formulated compound fertilizer (SCF) was applied together with commercial livestock manure (LSM) and urea. The treatment referred to as HCF received high rates of fertilizers (as common compound fertilizer, rapeseed cake manure and urea) to investigate the effect of over-application of fertilizers (Ni et al., 2019). The rates of fertilizers in treatments are presented in **Table 1**. RSM, SCF+RSM and RSM+LSM had the same total amounts of nutrient N, P and K, which were reduced by 25% compared to that of FP (**Table 1**). Compared to FP, N rate was increased (by 5.7%) in RSM, decreased in SCF+RSM (by 4.2%) and in SCF+LSM (by 19.5%). The ratio of N-P-K fertilizers was optimized to be close to their ratios in young shoots. The ratio was decreased in RSM, SCF+RSM and HCF whereas that of K was slightly increased in SCF+LSM compared to that of FP (**Table 1**). Organic manures and compound fertilizers were applied in October. Urea in SCF+RSM, SCF+LSM and HCF was separately into two applications with equal amounts, one in early February and the second in the end of April after the harvest of spring tea. Fertilizers were applied to furrows between rows followed by covering with soil. There were totally six treatments including a control (CK) without any fertilizers. The plot area was 20 m^2^ and plots were randomly arranged in the field. Each treatment was replicated for three times. All other field management was the same as in the FP.

**Table 1:** Source, amount and cost of fertilizers in treatments.

Nutrient	Treatment					
	CK	FP	RSM	SCF+RSM	SCF+LSM	HCF
Chemical fertilizers						
N (kg ha^-1^)	0	144	0	168	168	850
P (kg ha^-1^)	0	144	0	65	65	450
K (kg ha^-1^)	0	144	0	98	98	450
Organic fertilizers						
N (kg ha^-1^)	0	117	276	83	42	38
P (kg ha^-1^)	0	54	128	39	38	16
K (kg ha^-1^)	0	30	71	22	63	11
ONSR (%)	/	45	100	33	20	4
Sum						
N (kg ha^-1^)	0	261	276	250	210	888
P (kg ha^-1^)	0	86	56	43	46	203
K (kg ha^-1^)	0	145	58	97	134	383
Total (kg ha^-1^)	0	492	390	390	390	1474
N-P-K ratio	/	1-0.33-0.55	1-0.20-0.21	1-0.17-0.39	1-0.22-0.64	1-0.23-0.43
Cost (Yuan ha^-1^)	0	9840	14100	7995	7095	15105

CK, no fertilizer; FP, farmer’s practice consisting of common compound fertilizer (N-P_2_O_5_-K_2_O=15-15-15) and rapeseed cake manure; RSM, rapeseed cake manure; SCF+RSM, specially formulated compound fertilizer (SCF, N-P_2_O_5_-K_2_O-MgO=18-8-12-2) and rapeseed cake manure; SCF+LSM, SCF and livestock manure; HCF, high amount of common compound fertilizer and rapeseed cake manure; ONSR, the share of N from organic manures in the total amount of N fertilizers; Cost, including fertilizer and application costs.

### 2.2 Samples and measurements

Young spring shoots were harvested by hand and were weighed. The weights of each harvest were summed as the annual fresh yield. Yield data of 2018-2020 were presented in the present work whereas those of 2017 was not included to eliminate the turnover effect from farmer’s practice to the fertilization treatments. Samples of young shoots were collected to measure the concentrations of nutrients in harvested teas. The concentration of N in teas and mature leaf samples was determined by an elemental analyzer (Vario Macro Cube, Elementar Analysensysteme GmbH, Langenselbold, Germany) and those of P and K were determined by Inductive Coupled Plasma-Atomic Emission Spectrometer (iCAP™ 7400 ICP-OES, Thermo Fisher Scientific, USA) after digestion at 550°C and re-dissolved in dilute nitric acid. Mature leaves of tea plants were sampled on March 21, 2020, dried in an electric oven at 60 °C, and finely ground to determine the concentrations of nutrients.

The contents of quality-related metabolites in teas were determined in the fourth year of field experiment assuming that the nutrition status of plants reached stable conditions. Tea samples of the first and second harvest were taken on March 22 and 26, 2020, quickly frozen in liquid nitrogen, freeze-dried and finely ground. Powder of tea samples (100 mg) were extracted with 5 mL of H_2_O in a boiling water bath for 5 min (Ma et al., 2021). The extract was used for the determination of total free amino acid (TFAA) by spectrophotometry after reaction with ninhydrin reagent and total polyphenol (TP) after reaction with Fe-tartrate reagent (Ruan et al., 2013). The composition of free amino acids in the extract was determined by High Performance Liquid Chromatography (HPLC) using Waters AccQ•Tag™ pre-column derivatization kit following the manufacturer’s instruction (Waters Corporation, Milford, MA, USA). The content of caffeine and the composition of catechins in the extract were determined by HPLC (Waters Corporation, Milford, MA, USA) equipped with a C_18_ reverse phase column (250 × 4.6 mm) according to the method previously described (Wu et al., 2012). Contents of amino acids, caffeine and catechins were quantified by their areas of chromatographic peaks against those of authentic standards.

Soil samples were taken in the end of field experiment (October 2020) from 5 randomly selected sites to 1 m depth at five separate layers, i.e. 0-20, 20-40, 40-60, 60-80 and 80-100 cm and mixed thoroughly per plot. Stones and debris of roots were removed and samples were separated into two portions after thorough-out mixture. Fresh soil was temporarily stored in a refrigerator at 4°C before the determination of water content and inorganic nitrogen. Ammonium and nitrate in fresh soil were extracted by 2 mol l^-1^ potassium chloride and determined by Discrete Chemistry Analyzer (Smartchem 140, AMS Alliance, Frepillon, France). Soil pH was measured in 1:2.5 water paste of air-dried soil samples by a glass electrode (Orion 3 Star, Thermo Ltd., Waltham, MA, USA).

The average price of fresh spring teas was approximately 150 Yuan kg^-1^ according to the local market. The net profit of fertilization was calculated as the difference between the values of fresh young shoots of treatments (V_F_) and CK (V_CK_) and the fertilization cost (C_F_) according to the following Equation (1). Cost of harvesting young shoots was not taken into account as this provided important employment and cash income for local labors.


(1) $$\text{Profit}=\left({\text{V}}_{\text{F}}-{\text{V}}_{\text{CK}}\right)-{\text{C}}_{\text{F}}$$

Agronomical efficiency (AE, kg kg^-1^) was calculated from the yield (Y_F_) and the rate (R_F_) of N, P and K (**Table 1**) in the treatments relative to the yield of CK (Y_CK_) according to the following Equation (2):


(2) $$AE=\left(Y_{F}-{\text{Y}}_{\text{CK}}\right)/R_{F}$$

### 2.3 Estimation of greenhouse gas emission derived from fertilization

The greenhouse gas (GHG) emissions derived from fertilization were divided into four parts: production and transportation of fertilizers, and direct and indirect N_2_O emissions caused by N fertilizer application according to default method of the Intergovernmental Panel on Climate Change (IPCC) (Forster et al., 2007; Hergoualc’h et al., 2019). The area scaled GHG (CO_2_ equivalent) generated from the production and transportation of chemical fertilizers (GHG_A-Pr_ and GHG _A-Tr_) was estimated from their application rates per hectare (*F*) and respective emission factors (EF) according to the following Equation (3). The emission factors of manufacture and transportation were 8.21 and 0.09 for N fertilizer, 0.73 and 0.06 for P fertilizer, 0.5 and 0.05 for K fertilizer, respectively (Forster et al., 2007; Zhang et al., 2013).


(3) $${\text{GHG }}_{\text{A}-\text{Pr}}{\text{ or GHG }}_{\text{A}-\text{Tr}}=\sum_{i=N,P,K}^{3}{\text{EF}}_{\text{ i}}\times F_{i}$$

The direct emission of N_2_O (dE_N2O_) from the application of chemical and organic N fertilizers was estimated from their application rates per hectare (*F_N_*) and their respective emission factors according to the following Equation (4). The EF for chemical and organic N fertilizers were 0.0175 and 0.0261 according to the most recent meta-analysis (Wang et al., 2020a). These values were slightly lower than 0.0272 (or 2.72%) which used for both chemical and organic N fertilizers in the previous works (Tang et al., 2021).


(4) $${\text{dE }}_{\text{N}2\text{O}}=\;\sum_{i=organic\;N,\;chemical\;N}^{2}{\text{EF}}_{\text{i}}\times F_{Ni}$$

The indirect N_2_O emissions (idE_N2O_) caused by the application of N fertilizers were calculated from N rate per hectare, fractions of N loss through leaching (FN_L_, %) and runoff (FN_R_, %), and default emission factor (0.011, Hergoualc’h et al., 2019) according to the following Equation (5). FN_L_ was 14.5% based on our three-year lysimeter experiment of similar soil type and texture with the same cultivar Baiye-1 (Zheng, 2022). FN_R_ was 8.2% according to a field experiment of green tea with similar soil, weather and topographical conditions (Xie et al., 2021). The current value of FN_L_ was higher while that of FN_R_ was lower than 9.8% which was adopted for both in the previous work (Tang et al., 2021).


(5) $${\text{idE }}_{\text{N}2\text{O}}=F_{N}\times \left({\text{FN}}_{\text{L}}+{\text{FN}}_{\text{R}}\right)\times 0.011$$

The direct and indirect emissions of N_2_O (dE_N2O_) were converted to CO_2_ equivalent greenhouse gas emission (GHG_A-FN_) according to the following Equation (6).


(6) $${\text{GHG}}_{\text{A}-\text{FN}}=\left({\text{dE}}_{\text{N}2\text{O}}+{\text{idE}}_{\text{N}2\text{O}}\right)\times \frac{44}{28}\times 298$$

The area scaled total emission (GHG_A_) from production, transportation and application of fertilizer was calculated according to the following Equation (7) and was further converted to per yield (GHG_Y_) and profit (GHG_P_) scales according to the following Equations (8), (9), respectively.


(7) $${\text{GHG}}_{\text{A}}={\text{ GHG}}_{\text{A}-\text{Pr}}+{\text{GHG}}_{\text{A}-\text{Tr}}+{\text{GHG}}_{\text{A}-\text{FN}}$$

(8) $${\text{GHG}}_{\text{Y}}={\text{ GHG}}_{\text{A }}÷\text{Yield}$$

(9) $${\text{GHG}}_{\text{P}}={\text{ GHG}}_{\text{A }}÷\text{Profit}$$

### 2.4 Statistical analysis

To test the effect of fertilization data were subjected to one-way analysis of variance (ANOVA) combined with the least significant difference (LSD) test by SigmaStat embedded in SigmaPlot (Version 12.5, Systat Software Inc., Palo Alto, CA 94303).

## 3 Results

### 3.1 The concentrations of nutrients in teas and mature leaves of tea plants

**Figure 1:**
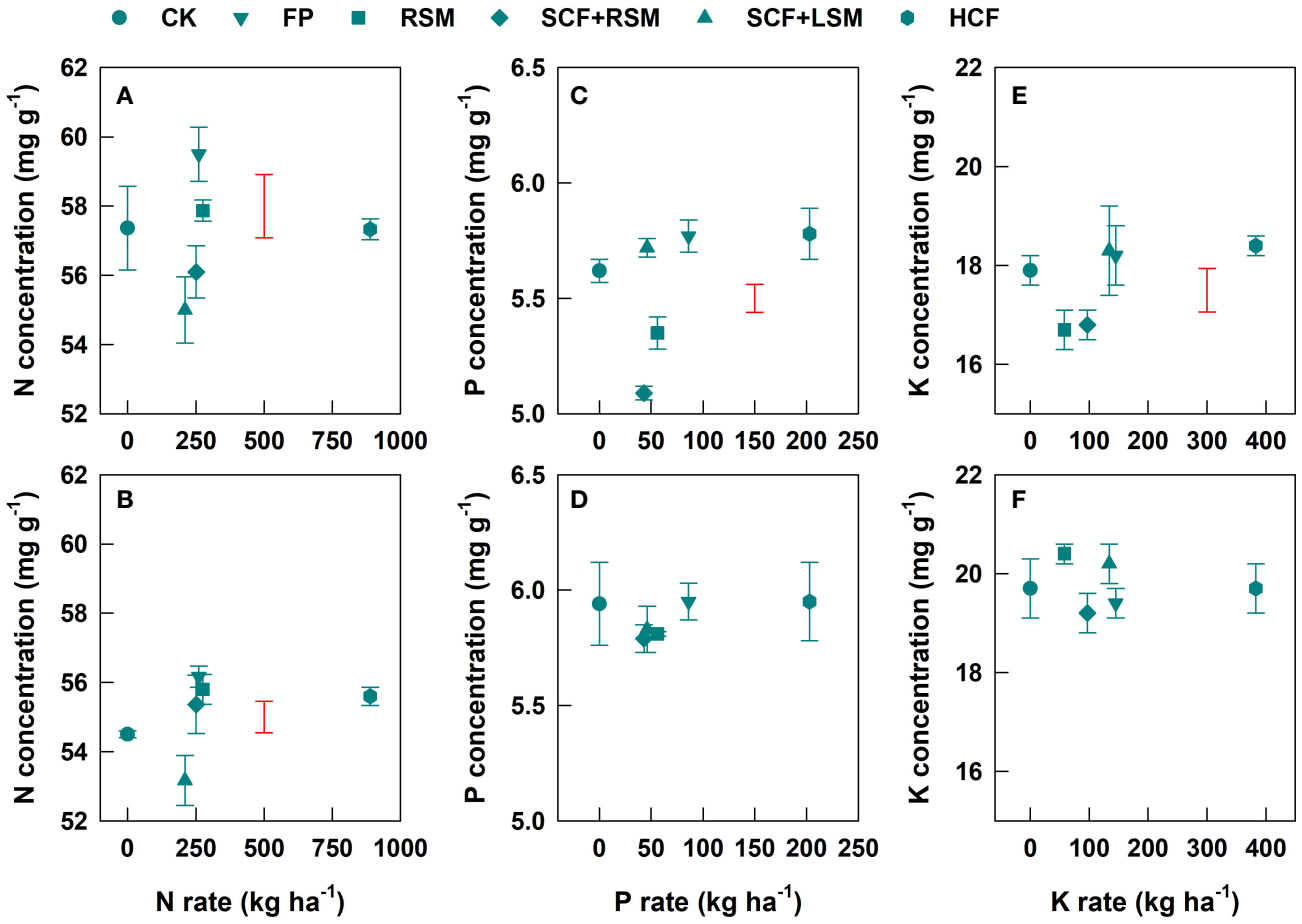
Response of the concentrations of N **(A, B)**, P **(C, D)** and K **(E, F)** in teas of the first harvest **(A, C, E)** and second harvest **(B, D, F)** to the application rate of fertilizers. Error bars are standard deviations of three replicates. Bars in red without data point are LSD values indicative of significant (p<0.05) difference among treatments.

The concentrations of N, P and K in teas of both harvests appeared not directly related to the application rates of fertilizers (**Figure 1**). However, the concentrations of N in teas of both harvests were significantly decreased in SCF+LSM (**Figures 1A, B**). The concentrations of P and K in teas were inconsistently affected by fertilization treatments between the two harvests. Their concentrations in the first harvest (March 22) were considerably decreased in RSM and SCF+RSM compared to those in CK (**Figures 1C, E**). Their concentrations in the second harvest were unaffected by fertilization treatments (**Figures 1D, F**).

**Figure 2:**
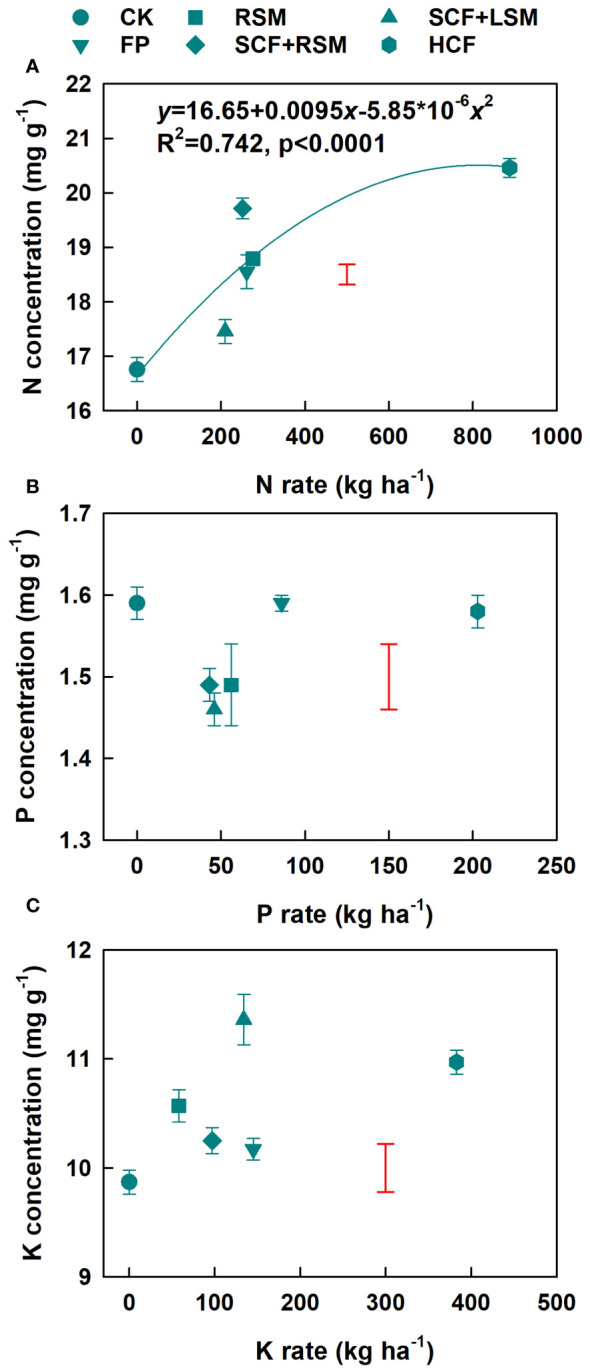
Response of the concentrations of N **(A)**, P **(B)** and K **(C)** in mature leaves of tea plants to the application rate of fertilizers. Error bars are standard deviations of three replicates. Bars in red without data point are LSD values indicative of significant (p<0.05) difference among treatments.

**Figure 3:**
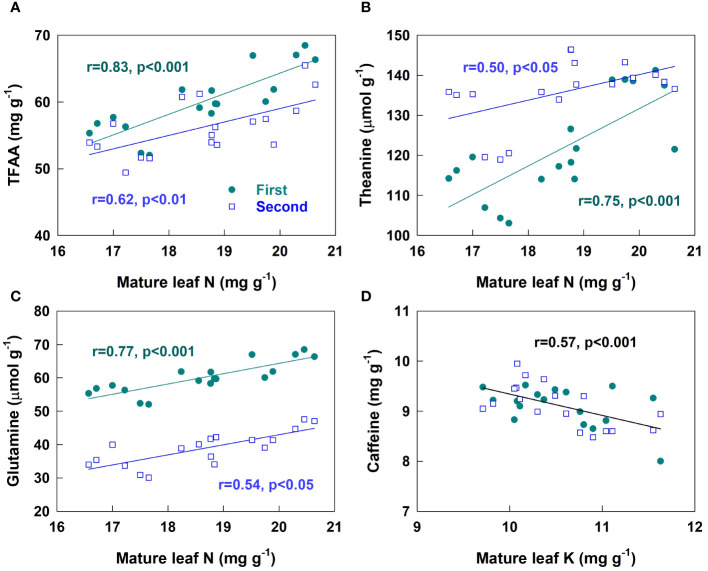
Relations between the concentrations of total free amino acid (TFAA, **A**), theanine **(B)**, glutamine **(C)** and caffeine **(D)** in teas of the first (closed symbols) and second harvest (open symbols) with those of N or K in mature leaves of tea plants.

Compared to CK, the concentrations of N in mature leaves of tea plants were significantly increased by fertilization. Their concentrations were most considerably increased in HCF and HCF+RSM but least increased in SCF+LSM (**Figure 2A**). There were significantly positive relation between the N concentration of mature leaves and the N application rate, which could be well described by a quadratic equation (R^2 =^ 0.742, p<0.0001). The concentrations of P in mature leaves of tea plants were significantly decreased in RSM, SCF+RSM and SCF+LSM and unchanged in FP and HCF compared to that of CK (**Figure 2B**). The concentrations of K in mature leaves of tea plants were significantly increased in SCF+LSM, HCF and RSM but were unaffected in SCF+RSM and FP compared to that CK (**Figure 3B**). The responses of P and K concentrations in mature leaves to the rates of P and K fertilizers, respectively could not be described by any defined equations.

### 3.2 The concentrations of free amino acids in teas

The concentrations total free amino acid (TFAA) in teas were significantly increased in HCF but decreased in SCF+LSM in both harvests compared to those of CK (**Table 2**). Compared to that of CK, the TFAA concentration in the first harvest was significantly increased in SCF+RSM. The TFAA concentrations in both harvests were not significantly affected in RSM and FP. There were significantly (p<0.01) linear positive correlations between TFAA concentrations in both harvests and the N concentrations in mature leaves of tea plants (**Figure 3A**).

**Table 2:** Contents of total free amino acid (TFAA, mg g^-1^) and free amino acids (μmol g^-1^) in teas.

Treatment	TFAA	Thea	Gln	Glu	Asp	Phe	Ser
First harvest (March 22)							
CK	56.6±1.2cd	116.7±2.7b	36.5±3.1b	16.3±1.3b	11.0±0.9b	7.0±0.2ab	3.4±0.3b
FP	60.2±1.4bc	117.7±3.8b	40.4±1.7b	15.9±0.9b	11.5±0.6b	6.9±0.3ab	3.1±0.2b
RSM	59.9±1.7bc	119.6±6.4b	37.4±3.9b	17.0±1.2b	10.8±0.8b	6.6±0.2bc	3.1±0.3b
SCF+RSM	63.0±3.6b	138.8±0.2a	40.6±1.3b	17.3±1.0b	11.5±0.7b	7.0±0.1a	3.5±0.2b
SCF+LSM	53.5±2.4d	104.8±2.0c	31.6±1.9c	16.6±1.2b	10.6±0.8b	6.4±0.1cd	3.3±0.2b
HCF	67.3±1.1a	133.4±10.5a	46.4±1.5a	22.1±1.0a	13.1±0.2a	6.2±0.2d	4.3±0.6a
Second harvest (March 26)							
CK	54.7±1.8bc	135.4±0.4c	28.9±0.6bc	16.5±0.6a	8.8±0.3ab	4.6±0.1b	3.9±0.1a
FP	58.5±4.3ab	135.8±1.9c	30.2±1.7b	15.9±1.5a	8.5±0.8ab	4.6±0.1b	3.8±0.3ab
RSM	55.1±1.1b	145.3±1.9a	27.3±3.0bcd	14.1±0.4b	7.7±0.1b	4.3±0.1c	3.4±0.2b
SCF+RSM	56.0±2.1b	140.1±2.8b	26.2±1.8cd	16.5±1.1a	8.9±0.7ab	5.6±0.0a	4.0±0.1a
SCF+LSM	50.9±1.3c	119.7±0.8d	24.5±1.0d	16.4±0.7a	8.6±0.4ab	4.6±0.1b	3.9±0.2a
HCF	62.2±3.4a	138.4±1.8bc	38.6±2.4a	17.0±0.6a	9.5±1.0a	4.2±0.2c	4.2±0.3a

Thea, theanine; Gln, glutamine; Glu, glutamate; Asp, asparate; Phe, phenylalanine.

Different letters following data of the same columns indicate significant differences (p< 0.05) among treatments for the specified harvest.

Data are means and standard deviations of three replicates.

The compositions of free amino acids were significantly affected by fertilizers but the effects were inconsistent between the two harvests (**Table 2**). Compared to CK, FP had little effect on the compositions of free amino acids. RSM affected the composition of free amino acids only in the second harvest, increasing that of theanine (Thea) but decreasing those of glutamate (Glu) and phenylalanine (Phe). SCF+RSM increased Thea concentrations in both harvests whereas had little effect on those of other amino acids. SCF+LSM decreased the concentrations of Thea and glutamine (Gln) in both harvests compared to CK. HCF increased concentrations of most amino acids including Thea compared to CK in the first harvest but had smaller effects in the second harvest. The concentrations of Thea and Gln of both harvests linearly related to the concentrations of N in mature leaves of tea plants (**Figures 3B, C**).

### 3.3 The concentrations of caffeine, total polyphenol and catechins in teas

Compared to CK, SCF+LSM decreased the concentrations of caffeine in both harvests (**Table 3**). SCF+RSM increased while HCF decreased the concentrations of caffeine in teas of the second harvest. The concentrations of caffeine was negatively correlated with K concentrations of mature leaves (**Figure 3D**). Compared to CK, SCF+RSM increased the concentrations of total polyphenol (TP) in both harvests mostly among the treatments. By contrast, HCF decreased the concentrations of TP, catechins and the ratio of TP/TFAA in both harvests compared to CK. The concentrations of TP and catechins in HCF were the lowest among fertilization treatments with only a few exceptions. The concentrations of catechins were also affected by other fertilization treatments but inconsistently between the two harvests (**Table 3**). SCF+LSM decreased the concentrations of EGC and ECG in the first harvest but increased those in the second harvest. FP decreased the concentrations of ECG in the first harvest and those of EGCG and EC in the second harvest. The concentrations of catechins were only weakly affected in RSM compared to CK.

**Table 3:** Concentrations of caffeine, total polyphenols (TP), the ratio of TP/TFAA and catechins in teas.

Treatment	Caffeine	TP	TP/TFAA	Catechin (mg g^-1^)			
				EGCG	EGC	ECG	EC
First harvest (March 22)							
CK	9.39±0.14a	238.1±5.6b	4.21±0.04ab	26.8±0.9a	9.77±0.23b	4.69±0.02a	4.26±0.07
FP	9.05±0.20ab	234.2±6.6bc	3.89±0.20b	24.9±0.2ab	9.87±0.20b	4.26±0.05b	4.07±0.09
RSM	9.15±0.36ab	238.4±2.2b	3.98±0.13b	26.4±1.1a	10.66±0.46a	4.79±0.26a	4.16±0.21
SCF+RSM	9.38±0.16a	249.9±7.6a	3.98±0.32b	26.2±0.5a	11.00±0.31a	4.70±0.12a	3.92±0.20
SCF+LSM	8.64±0.63b	235.2±3.3bc	4.40±0.20a	23.4±1.3bc	8.96±0.61c	4.05±0.31bc	3.94±0.14
HCF	9.10±0.36ab	226.3±1.7c	3.36±0.06c	22.4±1.5c	9.24±0.21c	3.93±0.09c	4.23±0.43
Second harvest (March 26)							
CK	9.23±0.22b	257.6±3.5ab	4.72±0.22a	24.9±0.9a	8.35±0.36b	3.80±0.06b	5.37±0.07a
FP	9.44±.20ab	238.9±4.0cd	4.10±0.27bc	22.7±0.5b	8.64±0.27b	3.86±0.18b	4.32±0.20d
RSM	9.08±0.19b	232.4±10.3d	4.22±0.11b	23.5±0.8ab	8.89±0.42b	3.68±0.09b	3.80±0.11e
SCF+RSM	9.66±0.32a	269.3±13.2a	4.81±0.41a	24.5±1.0a	8.71±0.27b	4.07±0.10a	4.52±0.12c
SCF+LSM	8.68±0.24c	249.4±5.4bc	4.90±0.09a	24.6±0.5a	10.070.38a	4.17±0.04a	5.08±0.08b
HCF	8.59±0.02c	230.9±4.2d	3.72±0.19c	17.6±0.4c	8.35±0.18b	3.49±0.02c	4.96±0.02b

EGCG, epigallocatechin-3-gallate; EGC, epigallocatechin; ECG, epicatechin-3-gallate; EC, epicatechin.

Different letters following data of the same columns indicate significant differences (p< 0.05) among treatments for the specified harvest.

Data are means and standard deviations of three replicates.

**Figure 4:**
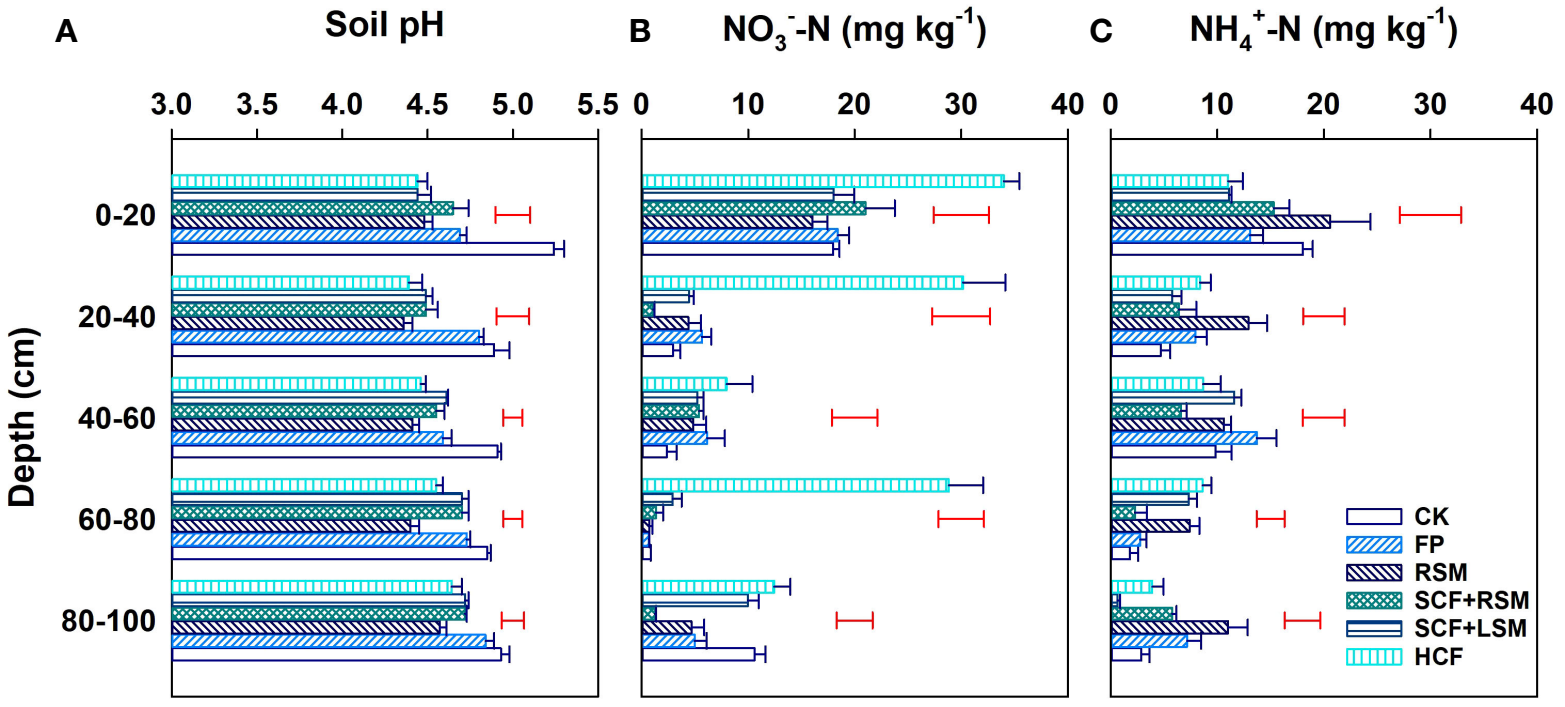
pH **(A)** and the contents of NH_4_^+^ **(B)** and NO_3_^-^ **(C)** in the soil profile. Error bars are standard deviations of three replicates. Single bars in red along with columns are LSD values indicative of significant (*p*<0.05) difference among treatments at the specified depth.

### 3.4 Yield, profit and nutrient use efficiency

The yield was relatively stable with small variations (CVs 7.7 − 12.1%) between experimental years (**Table 4**). Compared to CK without any fertilizers, fertilization treatments significantly increased tea yield by 33.6% − 58.7% (**Table 4**). Yields were slightly (p>0.05) higher in SCF+RSM, HCF and SCF+LSM and slightly lower in RSM than in FP. The profit of fertilization varied from 19794 to 51284 Yuan ha^-1^ (**Table 4**). The profit and profit/cost ratio were the lowest in RSM and the highest in SCF+RSM. SCF+RSM and SCF+LSM had 80% and 35% higher profits than FP, respectively. The HCF had higher profit than FP but much lower profit and profit/cost ratio than SCF+RSM and SCF+LSM.

**Table 4:** Fresh yield, profit, profit/cost ratio, nutrient content in young shoots and agronomical efficiency (AE).

Parameter	Treatment					
	CK	FP	RSM	SCF+RSM	SCF+LSM	HCF
Yield (kg ha^-1^)	673±82b	929±111a	899±77a	1069±76a	984±110a	1000±97a
Profit (Yuan ha^-1^)	/	28477	19794	51284	39443	33863
Profit/cost ratio	/	2.89	1.40	6.41	5.56	2.24
Nutrient content (kg ha^-1^)						
N	8.70±0.80d	12.37±1.63bc	11.89±0.66c	13.98±0.98ab	12.81±1.73abc	14.78±0.75a
P	0.89±0.10c	1.24±0.15b	1.20±0.06b	1.44±0.11a	1.33±0.14ab	1.51±0.10a
K	3.00±0.64c	4.18±0.90abc	4.09±0.62bc	5.06±0.82ab	4.47±0.97ab	5.43±0.21a
AE (kg kg^-1^)						
N	/	0.98±0.11b	0.82±0.10b	1.57±0.28a	1.48±0.16a	0.37±0.04c
P	/	2.96±0.34cd	4.05±0.51c	8.71±1.57a	6.90±0.76b	1.61±0.17d
K	/	1.77±0.20b	3.84±0.49a	4.00±0.72a	2.32±0.26b	0.85±0.09c

Different letters following data of the same line indicate significant difference (p< 0.05) among treatments.

Data are means and standard deviations of three years (2018-2020).

The contents of N, P and K in the young shoots, which were calculated from shoot dry tea yield and nutrient concentrations, were significantly increased by fertilization (**Table 4**). The contents of N and P were significantly increased, in a decreasing order, by HCF and SCF+RSM, followed by SCF+LSM, FP and RSM. Compared to CK, K contents of young shoots were increased significantly by HCF, SCF+RSM, SCF+LSM but unaffected by FP and RSM. The agronomic efficiency (AE) of N, P and K fertilizers were the highest in SCF+RSM and the lowest in HCF (**Table 4**).

### 3.5 Soil properties and greenhouse gas emissions

Fertilization significantly decreased soil pH of all layers (**Figure 4A**). The lowest soil pH was found in RSM and HCF. The concentrations of residual NO_3_^–^N and NH_4_^+^-N varied largely among different soil layers and mainly accumulated at the surface soil (depth 0-20 cm) (**Figures 4B, C**). The amounts of NO_3_^−^-N and NH_4_^+^-N in the surface soil (0-20 cm) accounted for 49.9% and 35.1% of the total profile (assuming an unform bulk density) down to 1 m depth, respectively. HCF had extremely high residual NO_3_^−^-N in the soil down to the depth of 60-80 cm. The concentration ratios of NO_3_^−^-N/NH_4_^+^-N were significantly higher in HCF than in other treatments.

Area (GHG_A_) and yield (GHG_Y_) scaled total greenhouse gas emissions were decreased in SCF+RSM, SCF+LSM and RSM compared to FP (**Table 5**). SCF+LSM had the lowest GHG_A_ and GHG_Y_. Compared to FP, the profit scaled total greenhouse gas emissions (GHG_P_) were decreased in SCF+RSM and SCF+LSM but increased in RSM. GHG_A_, GHG_Y_ and GHG_P_ were the highest in HCF, which were 3.7 − 4.4, 3.5 − 4.4 and 2.0 − 4.9 times higher than those in other fertilization treatments, respectively.

## 4 Discussion

### 4.1 Response of tea yield and quality to the rate and ratio of nutrients

Compared to CK, tea yield was significantly increased (33.6% − 58.7%) by fertilization, indicating the important contribution of fertilization to improve the productivity of Baiye-1. However, tea yields did not statistically differ among fertilization treatments in spite of different rates of fertilizers. The N concentrations of mature leaves and the contents of amino acids, TP and TP/TFAA significantly responded to the application rate of N fertilizers. In the present experiment, we included a special treatment HCF to evaluate the effect of extremely high rate of fertilizers which had been used in plantations of normal green cultivars (Mishima et al., 2010; Ni et al., 2019). High N rate in HCF significantly increased yield and the concentrations of TFAA and main amino acids (such as Thea and Gln) but decreased the concentrations of total polyphenol and catechins, resulting in the unbalance of TP and TFAA and weakening the intensity of the tea infusion. These results suggest that application N fertilizers at the rate of 250~276 kg ha^−1^ in SCF+RSM, FP and RSM appeared to supply sufficient N to tea plants. However, HCF produced higher profit regardless of higher fertilization cost than FP because of good price of tea. This might help explain the reason why over-application of fertilizers had been popular in tea plantations (Ni et al., 2019).

The contents of free amino acid and polyphenols were insignificantly affected in FP although the yield was significantly increased (by 38%) compared to CK. Compared to FP and CK, SCF+RSM increased the concentrations of TFAA, Thea, total polyphenol and catechins, thereby improved the overall tea quality. This was associated with the significantly increased concentration of N in mature leaves of tea plants in SCF+RSM (**Figure 2A**). Our previous work showed that N in the mature leaves is removable to support the growth and metabolism of spring tea hence significantly affects the contents of amino acids of spring tea (Liu et al., 2016), which is also supported by the present findings of their close relationships (**Figure 3**). The photosynthesis rate was likely improved in leaves of increased N content (Okano et al., 1997). SCF+RSM had slightly lower N rate than FP and it was likely that their different effects on tea quality might be partially related to the optimization of P, K rates and their ratio against N to better meet the requirement of tea plants. The amounts of P and K input in SCF+RSM were reduced by 47% and 31%, respectively, compared to FP, by replacing compound fertilizer of equal nutrient formular (N-P_2_O_5_-K_2_O = 15-15-15) with that of specialized formular (N-P_2_O_5_-K_2_O = 18-8-12). The ratio of N, P and K in SCF+RSM was optimized to 1-0.17-0.39, which was more closed to the ratio of their contents in young shoots (1-0.10-0.35, **Table 4**). This result is consistent with recent findings showing that over application of P and K fertilizers reduce green tea quality (Wei et al., 2022; Zhang et al., 2023). The concentrations of P and K in young shoots and mature leaves hardly responded to the rates of fertilizers (**Figures 1**, **2**). The specially formulated compound fertilizer contained Mg and provided additional Mg (16 kg MgO ha^-1^), which might also play a role (Ruan et al., 2012; He et al., 2023). A recent field experiment showed that application of tea-specific fertilizer together with rapeseed cake manure improves the aroma of green tea (Huang et al., 2022).

### 4.2 Response of tea yield and quality to the substitution of chemical fertilizers with organic manures

In the present work, chemical fertilizers were substituted by organic manures fully in RSM and partially in SCF+RSM and SCF+LSM. The yield increase and profit were much lower in the full substitution (RSM) than in the partial substitution (SCF+RSM, SCF+LSM) and even lower than those in FP for the high fertilization cost and relative lower yield of RSM. On the other hand, the quality of tea was improved in RSM and SCF+RSM for the significant increase of Thea content compared to in FP and CK. Nevertheless, it appeared that tea quality was improved more considerably in SCF+RSM relative to RSM for greater increase of the contents of amino acids (Glu, Phe and Ser), caffeine, TP and EGCG. These results confirmed our previous findings that partial substitution of chemical fertilizers by organic manure had better effect than the full substitution (Ji et al., 2018; Ma et al., 2022). These effects might be explained by the increased N concentration of mature leaves indicative of improved N nutrition of tea plants as the result of more readily available N supply in the partial (SCF+RSM) than in the full substitution (RSM) (**Figure 2A**).

The concentrations of TFAA and free amino acids in SCF+LSM were generally decreased compared to other fertilization treatments. Tea plants in SCF+LSM had low N status which was indicated by low N concentration in mature leaves of plants (**Figure 2A**). This is partly explained by its low N application rate, which was 16 − 24% lower than in FP, RSM and SCF+RSM. Furthermore, it was likely that the availability of N in livestock manure with a high C/N ratio was lower than that of rapeseed cake manure with a low C/N ratio. Previous work showed that there was a significant negative correlation between manure C:N ratio and N mineralization in the manure-amended soils (Qian and Schoenau, 2002). On the other hand, SCF+LSM decreased the concentrations of TFAA and main amino acids (Thea and Gln) compared to CK, which likely had been caused by a dilution effect as the result of significantly increased yield. The SCF+LSM scheme may be further optimized by increasing N application rate to improve N supply. The present work suggests that the effect of substitution is dependent on the type of organic manure and the bioavailability of nutrients.

### 4.3 Effect of the optimized nutrient management on nutrient use efficiency and environmental risks

Greenhouse gas emission in tea field has been estimated by IPCC default method in recent works. Production and application of N fertilizer accounted for a large portion of the GHG emissions in tea industry (Liang et al., 2021; Tang et al., 2021). SCF+LSM had the lowest area scaled GHG_A_ emission for its low N fertilizer rate. RSM had lower GHG_A_ than SCF+RSM and both of them had lower GHG_A_ than FP. However, an opposite result was found in greenhouse gas emissions (GHG_Y_ and GHG_P_) per yield and profit, which were much higher in RSM than in SCF+RSM and SCF+LSM. In all these treatments, it was assumed that organic manure had been locally recycled without long-distance transportation. In case of long-distance transportation of organic manures, RSM would have higher gas emissions due to its low nutrient content and high moisture content. Therefore, it appears that the full substitution of chemical fertilizers by rapeseed cake manure had not economic and sustainability advantages over the partial substitution. The application rate of N and the estimated GHGs in the present work were lower than those of the previous work in normal green-leaf cultivars based on Nutrient Expert (Tang et al., 2021).

Nutrient use efficiency is an important indicator evaluating the impact of an applied fertilizer on crop production and economic return (Fixen et al., 2015). A recent work showed that the agronomic efficiency (AE) was positively correlated with the free amino acid content, income, and negatively with global warming potential of tea (Tang et al., 2023). The present work showed that AEs were increased in SCF+RSM together with increases of tea productivity, the contents of free amino acids, total polyphenols and economic profit as well as reduced greenhouse gas emissions. The AEs of the present work were relatively low compared to the reported values in tea (Tang et al., 2023) and other crops (Fixen et al., 2015), which was likely attributed to the low yield of Baiye-1. On the other hand, AEs in HCF were decreased because of high amount of applied fertilizers. However, the low AEs of HCF did not necessarily reflect low income and low contents of free amino acids, a finding inconsistent with the recent finding (Tang et al., 2023). However, HCF remarkably increased greenhouse gas emission as well as the contents of residual NO_3_^−^-N in the deep soil hence enhanced the risk of N leaching. These results indicated that over-use of fertilizers deteriorated Baiye-1 tea quality and imposed serious environmental risks.

**Table 5:** Estimated greenhouse gas emission (CO_2_ equivalent) derived from fertilization.

Parameter	Unit	Treatment					
		CK	FP	RSM	SCF+RSM	SCF+LSM	HCF
GHG_A-Pr_	(kg ha^-1^)	0	1359	0	1476	1476	7532
GHG_A-Tr_	(kg ha^-1^)	0	29	0	24	24	126
Direct emission							
GHG_N_CF_	(kg ha^-1^)	0	1180	0	1377	1377	6966
GHG_N_OF_	(kg ha^-1^)	0	1430	3373	1014	513	464
Sum	(kg ha^-1^)	0	2610	3373	2391	1890	7430
Indirect emission							
Leaching	(kg ha^-1^)	0	195	207	188	157	665
Runoff	(kg ha^-1^)	0	110	117	106	89	375
GHG_A_	(kg ha^-1^)	0	4304	3696	4185	3636	16128
GHG_Y_	(kg kg^-1^)	0	4.63	4.11	3.92	3.70	16.13
GHG_P_	(kg Yuan^-1^)	0	0.151	0.187	0.082	0.092	0.476

GHG_A-Pr_ and GHG_A-Tr_, area scaled greenhouse gas emission derived from the production and transportation of chemical fertilizers, respectively; GHG_N_CF_ and GHG_N_OF_, area scaled greenhouse gas (N_2_O) emission derived from the application of chemical N and organic fertilizers, respectively; GHG_A_, area scaled total greenhouse gas emission derived from fertilization; GHG_Y_, yield scaled greenhouse gas emission; GHG_P_, profit scaled greenhouse gas emission.

## 5 Conclusion

In conclusion, the yield, quality and profit of albino tea were considerably increased by the optimization of nutrient management schemes formulating the rates and ratio of N, P, K and Mg and the partial substitution of chemical fertilizers with rapeseed cake manure. Partial substitution of chemical fertilizers with commercial livestock manure decreased the contents of free amino acids regardless of increasing yield, suggesting that the effect of substitution is dependent on the type of organic manure and the bioavailability of nutrients. Full organic substitution of chemical fertilizers increased tea yield and quality but had relatively low agronomic efficiency and profit than partial substitution. The effect of optimized nutrient management schemes was associated with the improvement of nutritional status in tea plants. The present work demonstrated that optimization of nutrient management considerably improved albino tea yield, quality and profit while decreased the input amounts of fertilizers and the intensity of greenhouse gas emissions.

## Data availability statement

The original contributions presented in the study are included in the article/supplementary material. Further inquiries can be directed to the corresponding author.

## Author contributions

YZ: Formal analysis, Investigation, Writing – original draft. LM: Conceptualization, Formal analysis, Funding acquisition, Investigation, Writing – original draft. SG: Formal analysis, Investigation, Writing – original draft. JR: Conceptualization, Data curation, Funding acquisition, Project administration, Supervision, Writing – original draft, Writing – review & editing.
